# Two new species of *Neofoleyellides* (Nematoda: Onchocercidae) parasitising anuran amphibians in South Africa

**DOI:** 10.1016/j.ijppaw.2021.02.018

**Published:** 2021-03-18

**Authors:** Yuriy Kuzmin, Edward C. Netherlands, Louis H. du Preez, Roman Svitin

**Affiliations:** a I.I. Schmalhausen Institute of Zoology NAS of Ukraine, 15 B. Khmelnytskogo str., 01030, Kyiv, Ukraine; b African Amphibian Conservation Research Group, Unit for Environmental Sciences and Management, North-West University, Private Bag X6001, Potchefstroom, 2520, South Africa; c South African Institute for Aquatic Biodiversity, Somerset Street, Machanda, 6140, South Africa

**Keywords:** Nematode, *Neofoleyellides*, Amphibia, Anura, *Amietia*, *Leptopelis*, Limpopo, KwaZulu-natal, South Africa

## Abstract

The genus *Neofoleyellides* was recently erected for a single species, *Neofoleyellides boerewors* from bufonid hosts in South Africa. In present study, we discovered two undescribed species of *Neofoleyellides*, namely *N. steyni* n. sp. and *N. martini* n. sp. parasitising frogs *Amietia delalandii* and *Leptopelis natalensis*, respectively. Both species differ from *N. boerewors* and between each other in shape and relative length of oesophagus, size of spicules, arrangement of genital papillae and morphology of caudal alae in males. Phylogenetic analysis based on concatenated fragments of the 18S ribosomal ribonucleic acid (18S rRNA) and the Cytochrome oxidase *c* subunit I (COI) genes confirmed both species as *Neofoleyellides* sister to Icosiellinae and Oswaldofilariinae.

## Highlights

• Two new species of *Neofoleyellides* described from frogs *Amietia delalandii* and *Leptopelis natalensis*.
• Morphological characters of *N. boerewors*, *N. steyni* n. sp. and *N. martini* n. sp. compared.
• Phylogenetic tree based on 18S rDNA and COI mtDNA sequences generated.
• Morphological and phylogenetic characteristics of three *Neofoleyellides* spp. discussed.

## Graphical abstract

**Figure:**
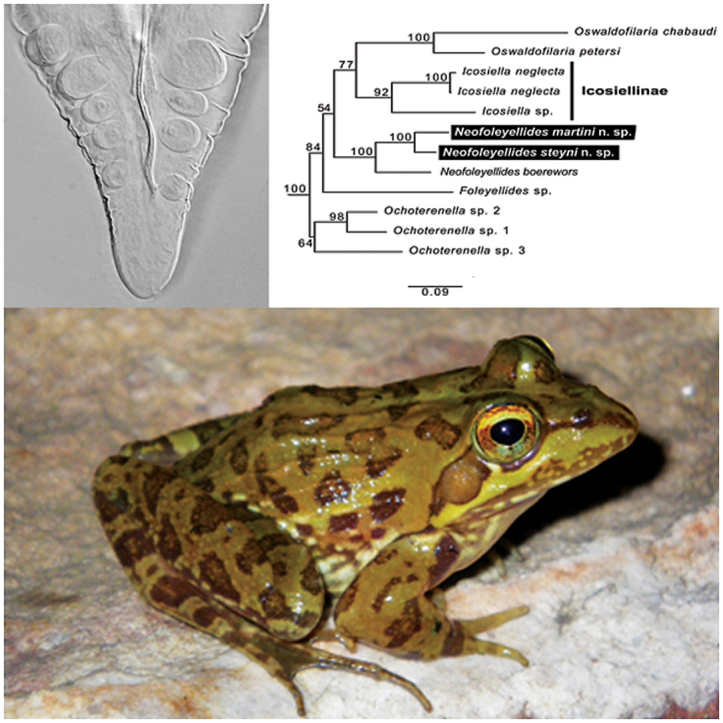

## 1 Introduction

Filarial nematodes parasitising amphibians are known from all continents, excluding Australia (Lefoulon et al., 2015). This group presently consists of 42 species assigned to six genera within the family Onchocercidae (Anderson et al., 2009). The majority of species are reported from Asia and the Americas, with four species from Madagascar and only two species reported from continental Africa. The first of them, *Foleyellides duboisi* (Gedoelst, 1916) was described from an unidentified frog in Democratic Republic of the Congo (Gedoelst, 1916). The second one was recently described as *Neofoleyellides boerewors* Netherlands, Svitin, Smit et Du Preez, 2020 from the toads (Bufonidae) *Sclerophys gutturalis* (Power, 1927) and *S. garmani* (Meek, 1897) in northern KwaZulu-Natal, South Africa (Netherlands et al., 2020).

Phylogenetic relationships of onchocercid filarial nematodes were recently investigated based on of seven loci (two mitochondrial and five nuclear genes) of 48 species belonging to seven subfamilies (Lefoulon et al., 2015). The study by Lefoulon et al. (2015) included four species parasitic in amphibians: *Icosiella neglecta* (Diesing, 1851) and three unidentified species of *Ochoterenella* Caballero, 1944, monophyletic with two species of *Oswaldofilaria* Travassos, 1933 parasitic in reptilian hosts. Subsequently, Netherlands et al. (2020) compared the phylogenetic relationships of amphibian onchocercids based on 18S rDNA and COI gene sequences, including two additional species from amphibian hosts to their analysis, *N. boerewors* and *Foleyellides* sp. In both studies, all species parasitising amphibians were recovered as the most distantly related genera of the family tree, however, relationships between *Foleyellides*, *Neofoleyellides* and *Ochoterenella* remained unresolved.

During a recent parasitological survey of amphibians in the Limpopo and KwaZulu-Natal Provinces of South Africa, numerous filarial nematodes were collected from common river frogs, *Amietia delalandii* (Duméril et Bibron, 1841) and Natal forest tree frogs *Leptopelis natalensis* (Smith, 1849). Based on the morphology of apical extremity, nematode specimens conform to species of *Neofoleyellides*. Specimens from both host species appeared to be different from each other and from *N. boerewors* and, therefore, are described herein as two new species, *N. steyni* n. sp. and *N. martini* n. sp. Furthermore, phylogenetic relationships of both new species were analysed based on nuclear 18S rRNA and mitochondrial COI genetic markers.

## 2 Materials and methods

Amphibians were collected using active sampling from two distant localities in South Africa. *Amietia delalandii* were collected from two sites in the vicinity of Louis Trichardt, Limpopo Province in February 2019. *Leptopelis natalensis* were collected from a single site, namely Alfred Park in Pinetown, KwaZulu-Natal Province in January 2020. All 17 collected specimens of *A. delalandii* were dissected at field workstation. Blood was collected, screened and stained following standard methods (Netherlands et al., 2015). Forty-three specimens of *L. natalensis* were screened for microfilariae, of which 14 were transported back to the North-West University (NWU) African Amphibian Conservation Research Group laboratory (Potchefstroom, North-West Province, South Africa) for further examination. Of the 14 specimens transported back eight had microfilariae in blood (positive with filariaemia) and five contained adult filariae visible under the frog's skin, an additional specimen negative for microfilaria or adult worms was included.

Before dissection, frogs were anaesthetised in 6% ethyl-3-aminobenzoate methanesulfonate (MS222) (Sigma-Aldrich Co., St. Louis, Missouri, USA) and subsequently euthanized according to the approved standard operation procedure (SOP) of the North-West University AnimCare Ethics Committee (ethics number: NWU-00492-16-S5). Adult filarial nematodes were removed from the body cavity, washed in saline, fixed in hot 70% ethanol and subsequently stored in 70% ethanol. Prior to microscopic examination, the nematodes were placed in distilled water for about 30 min and then cleared in lactophenol. The morphology of the nematodes was studied, and photomicrographs were taken using ZEISS axio ZII, Nikon AZ100 and Nikon ECLIPSE *Ni* compound microscopes. In total, 142 adult nematodes were examined, of which 60 were measured. Mean value, standard deviation (SD) and coefficient of variation (CV) were calculated for each morphometric character. All measurements in the text are given in micrometres unless otherwise indicated and presented as ranges followed by measurements of holotype or allotype in square brackets. Tukey HSD test for selected morphometric characters between different species was performed in SPSS software.

For molecular work, mid-body fragments of two males and one female paratypes of *N. steyni* n. sp. and two male and three female paratypes of *N. martini* n. sp. were used. DNA was extracted using the PCRBio Express Extraction Kit following the manufacturer's instructions. Cytochrome oxidase c subunit I (COI) amplicons were obtained using the primer pair COIintF (5′-TGA TTG GTG GTT TTG GTA A-3′) and COIintR (5′-ATA AGT ACG AGT ATC AAT ATC-3′). Ribosomal small subunit (18S rDNA) amplicons were obtained using the primer pair F18ScF1 (5′-ACC GCC CTA GTT CTG ACC GTA AA-3′) and F18ScR1 (5′-GGT TCA AGC CAC TGC GAT TAA AGC-3′). The thermocycling profile was as follows: 2 min at 94 °C for denaturation, 40 cycles of at 95 °C for 30 s, 52 °C for 30 for COI or 58 °C for 18S rDNA and 72 °C for amplification, following the final extension at 72 °C for 10 min (Lefoulon et al., 2015). PCR products from each sample were sent to a commercial sequencing company (Inqaba Biotechnical Industries (Pty) Ltd, Pretoria, South Africa) for purification and sequencing in both directions. Resultant sequences were assembled, and chromatogram-based contigs were generated and trimmed using Geneious Prime software (www.geneious.com).

For phylogenetic analysis, concatenated 18S rRNA and COI gene sequences were aligned, using MUSCLE (Edgar, 2004), with different representative sequences downloaded from GenBank and to the sequences generated in the current study (Table 1). Following Lefoulon et al. (2015), *Filaria latala* Chabaud & Mohammad, 1989 was selected as the outgroup. The GBlocks server was used to remove any alignment gaps and ambiguities, selecting the parameters to allow for smaller final blocks with gap positions (Castresana, 2000, Talavera and Castresana, 2007). To infer phylogenetic relationships a partitioned Maximum likelihood (ML) analysis was performed using RAxML version 8 (Stamatakis, 2014), implemented from within Geneious Prime. For the ML analysis, nodal support was assessed using 1000 rapid bootstrap inferences and partitioned according to the 18S rRNA (1–662 nt) and COI (660–1293 nt) gene fragments. The evolutionary model was estimated, evaluated, and optimized by RAxML version 8.2.11 implemented from within Genious Prime under the General Time Reversible model, with a proportion of invariable sites, and discrete gamma distribution (GTR + I + Γ).

**Table 1:** Summary of filarial species, their host and accession numbers, used in phylogenetic analyses in this study.

Subfamilies	Species	Hosts	Accession numbers	
			18S	COI
Icosiellinae	*Icosiella neglecta* (Diesing, 1851)	*Pelophylax ridibunda*	KP760137	KP760188
	*I. neglecta*	*Pelophylax* kl*. esculeta*	KP760138	KP760189
	*Icosiella* sp.	*Conraua goliath*		MH182623
Oswaldofilariinae	*Oswaldofilaria petersi* Bain and Sulahian 1974	*Crocodilurus amazonicus*	KP760160	KP760205
	*Oswaldofilaria chabaudi* Pereira, Souza and Bain, 2010	*Tropidurus torquatus*	KP760159	KP760204
Waltonellinae	*Foleyellides* sp.	*Rana pustulosa*		KC130675
	*Ochoterenella* sp. 1	*Rhinella granulosa*	KP760151	KP760198
	*Ochoterenella* sp. 2	*Rhinella marina*	KP760152	KP760199
	*Ochoterenella* sp. 3	*Phyllomedusa bicolor*	KP760150	KP760197
	*Neofoleyellides boerewors*	*Sclerophrys gutturalis*	MN689251	MN663133
	*Neofoleyellides martini* n. sp.	*Leptopelis natalensis*	MW599275	MW774895
	*Neofoleyellides steyni* n. sp.	*Amietia delalandii*	MW599274	MW598467
Filarioidea (outgroup)	*Filaria latala* Chabaud and Mohammad, 1989	*Panthera leo*	KP760135	KP760186

## 3 Results

In total, 142 specimens of *N. steyni* n. sp. and 122 specimens of *N. martini* n. sp. were recovered from 12 infected *A. delalandii* and eight *L. natalensis*, respectively. Most adult nematodes were found in the body cavity, while several specimens were collected subcutaneously, in the pericardium and between the hip muscles.

### 3.1 Species descriptions

**Family Onchocercidae** Leiper, 1911

**Subfamily Waltonellinae** Bain et Prod’Hon, 1974.

**Genus *Neofoleyellides*** Netherlands, Svitin, Smit et Du Preez, 2020.

*Neofoleyellides steyni* n. sp.

*Type-host*: *Amietia delalandii* (Duméril et Bibron, 1841) (Amphibia: Anura: Pyxicephalidae).

*Site of infection*: body cavity, subcutaneous.

*Type-locality*: Louis Trichardt, Limpopo Province, South Africa. Coordinates: 23°05′32.6″S, 30°07′26.5″E.

Type-specimens: Holotype (NMB P742), allotype (NMB P743) and 36 paratypes (NMB P744–745), deposited in the Parasitic Worm Collection, National Museum, Charles Street, Bloemfontein, South Africa.

*Infection parameters*: Intensity 1–54 (mean 11.8); prevalence 71%; mean abundance 8.4.

*Representative DNA sequences*: 18S [MW599274], COI [MW598467].

*Etymology*: The species named after Dr Koos Steyn who provided tremendous support with sample collection.

*ZooBank registration*: To comply with the regulations set out in article 8.5 of the amended 2012 version of the International Code of Zoological Nomenclature (ICZN, 2012), details of the new species have been submitted to ZooBank. The Life Science Identifier (LSID) for *Neofoleyellides steyni* n. sp. is urn:lsid:zoobank.org:act:[228104A4-D93C-4D89-B343-FB337D8B9E5B].

**Fig. 1:**
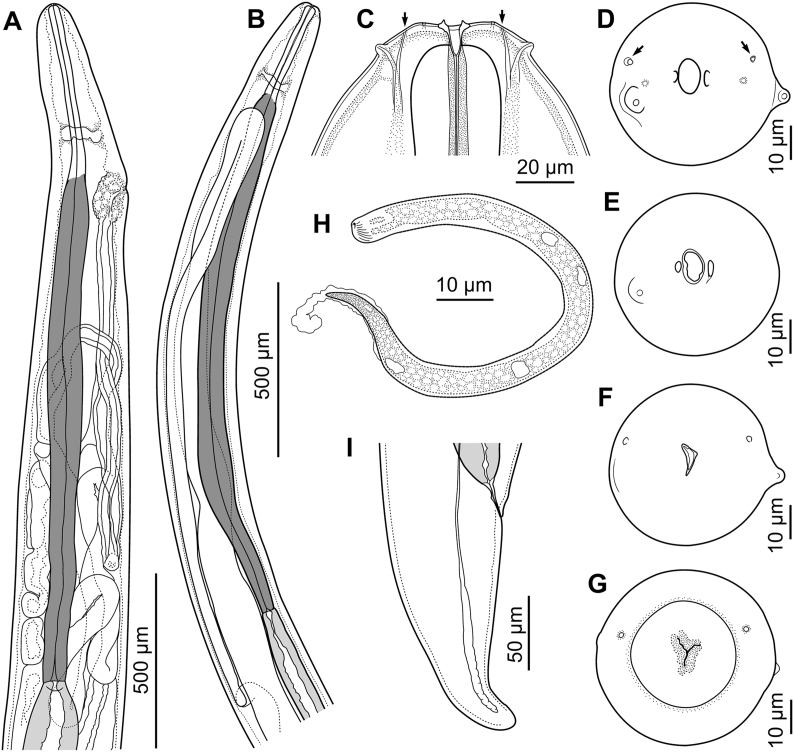
*Neofoleyellides steyni* n. sp. from *Amietia delalandii* (Duméril et Bibron, 1841), line drawings. A – fragment of body at anterior end, female, lateral view; B – fragment of body at anterior end, male, lateral view; C – anterior extremity, female, lateral view; D–G – anterior extremity, female, apical view, optical sections at different depth of focus; H – microfilaria; I – posterior end of body, female, lateral view.

**Fig. 2:**
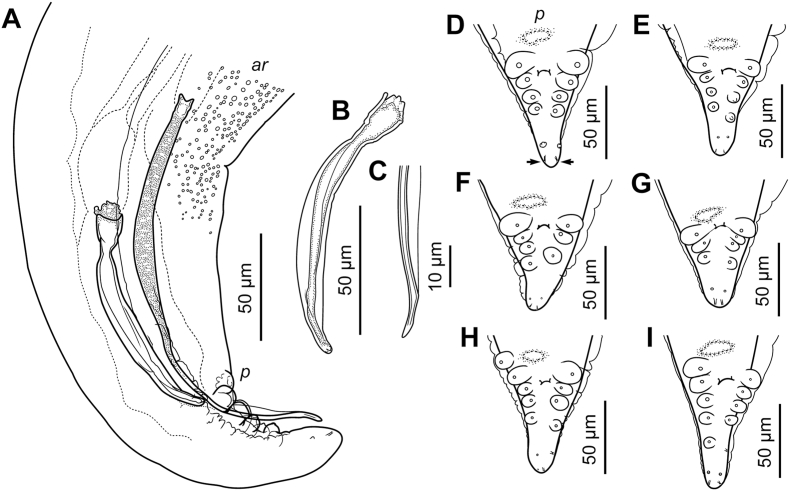
*Neofoleyellides steyni* n. sp. from *Amietia delalandii* (Duméril et Bibron, 1841), line drawings. A – posterior end of body, male, lateral view; B – right spicule, lateral view; C – distal end of the left spicule, lateral view; D–I – posterior end of body, male, ventral view, variations of the arrangements of caudal papillae.

**Fig. 3:**
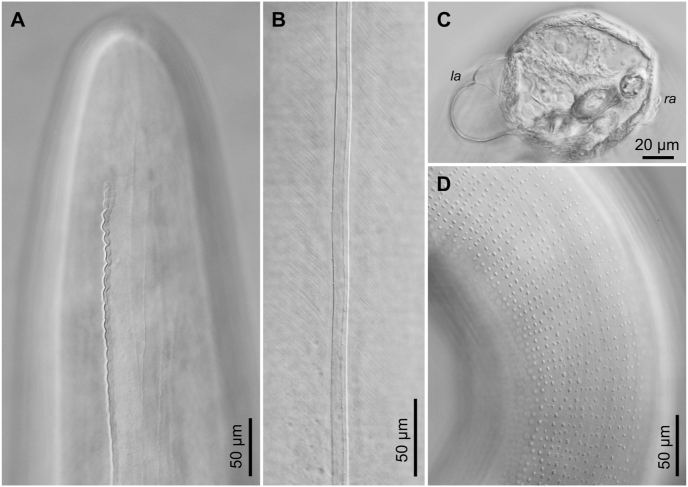
*Neofoleyellides steyni* n. sp. from *Amietia delalandii* (Duméril et Bibron, 1841), photomicrographs. A–C – lateral alae, male: A – anterior end, B – mid-body level, C – transverse section at level of posterior end, la – left ala, ra – right ala; D – area rugosa.

Description (Fig. 1, Fig. 2, Fig. 3).

General. Body thread-like, tapering on both extremities (Fig. 1, Fig. 2A). Females about 2–3 times longer than males. Anterior end rounded. Body cuticle finely transversely striated. Lateral alae present in both sexes, beginning at level of mid-length of muscular oesophagus (Fig. 3A), almost reaching tail tip posteriorly. Alae narrow (Fig. 3B), triangular in transverse sections along most of body length, slightly inflated and folded in anterior and posterior regions in females (Fig. 1, Fig. 3A), more prominently inflated in caudal region of males.

Oral opening oval, elongated dorso-ventrally (Fig. 1D). Two small lateral parastomal structures beside oral opening. One pair of large cephalic papillae with rounded tips located sub-laterally, slightly shifted to ventral side; one pair of smaller papillae, flattened and less distinct, located laterally closer to oral opening; amphidial openings pore-shaped, distinct, sub-lateral in position, slightly shifted to dorsal side (Fig. 1D). Buccal capsule small, cuticularised, wider anteriorly, dorso-ventrally elongated in apical view (Fig. 1C, E, F).

Muscular oesophagus comparatively short and narrow, rounded on anterior end (Fig. 1C), widening posteriorly, with slight constriction in posterior part, just anterior to junction with glandular oesophagus (Fig. 1A and B). Lumen of muscular oesophagus tri-radiate (Fig. 1G). Border between glandular and muscular oesophagus indistinct. Glandular oesophagus wider and approximately 3–4 times longer than muscular oesophagus, its anterior half gradually widening posteriorly, posterior half narrowing (Fig. 1A and B). Nerve ring surrounding constriction in posterior part of muscular oesophagus.

Males (30 specimens) Table 2. Body 17–26 [19] mm long, 130–209 [142], 217–316 [217] and 203–312 [235] wide at nerve ring, oesophageal-intestinal junction and mid-body, respectively. Posterior part of body corkscrew-shaped. Lateral alae beginning at 50–166 [85] from anterior end. Buccal capsule 4–7 [4] long, 5–10 [7] wide. Muscular portion of oesophagus 219–507 [270] long, 26–48 [27], 26–49 [35] and 22–54 [52] wide at anterior, mid-length and posterior level, respectively. Glandular portion of oesophagus 1032–1813 [1125] long, 29–75 [67], 80–155 [99] and 48–88 [60] wide at anterior, mid-length and posterior level, respectively. Total length of oesophagus 1318–2217 [1395], or 6.3–11.9 [7.5] % of body length. Nerve ring located at 167–351 [245] from anterior end; this distance corresponding to 10.7–19.4 [17.6] % of total oesophagus length. Anterior part of testis narrow, forming loops and bends in region of glandular oesophagus (Fig. 1B). Posterior to oesophago-intestinal junction, testis wide and straight. Ejaculatory duct narrower than testis, with funnel-shaped proximal part.

**Table 2:** Metric characters of males of *Neofoleyellides* spp. from South-African amphibians; measurements are presented as mean ± SD and coefficient of variation (in %) in parentheses.

Character	*N. steyni* n. sp. (N = 30)	*N. martini* n. sp.(N = 30)	*N. boerewors* (after Netherlands et al., 2020)
Body length, mm	20 ± 2 (9)	17 ± 1 (7)	22 ± 3 (14)
Body width at nerve ring	159 ± 16 (10)	128 ± 9 (7)	120 ± 12 (10)
Body width at oesophago-intestinal junction	259 ± 21 (8)	235 ± 16 (7)	190 ± 22 (12)
Body width at mid-body	264 ± 24 (9)	255 ± 20 (8)	202 ± 29 (15)
Distance from anterior extremity to anterior end of lateral alae	110 ± 33 (30)	113 ± 35 (31)	not measured
Length of buccal capsule	5 ± 1 (18)	–	4 ± 1 (20)
Width of buccal capsule	7 ± 1 (18)	–	9 ± 2 (18)
Length of muscular oesophagus	307 ± 64 (21)	250 ± 24 (10)	282 ± 42 (15)
Muscular oesophagus width in anterior part	36 ± 5 (13)	33 ± 3 (10)	38 ± 6 (16)
Muscular oesophagus width at mid-length	38 ± 6 (15)	36 ± 5 (13)	34 ± 5 (16)
Muscular oesophagus width in posterior part	39 ± 7 (19)	33 ± 4 (13)	41 ± 6 (14)
Length of glandular oesophagus	1355 ± 223 (16)	1252 ± 124 (10)	918 ± 146 (16)
Glandular oesophagus width in anterior part	53 ± 10 (18)	56 ± 9 (15)	66 ± 12 (18)
Glandular oesophagus width at mid-length	117 ± 18 (16)	100 ± 13 (13)	106 ± 23 (22)
Glandular oesophagus width in posterior part	64 ± 11 (17)	76 ± 12 (53)	96 ± 27 (28)
Total length of oesophagus	1663 ± 253 (15)	1501 ± 128 (9)	1204 ± 172 (14)
Total length of oesophagus, % of body length	8.1 ± 1.3 (16)	9.0 ± 1.0 (11)	5.6 ± 0.6 (12)
Distance to nerve ring	248 ± 34 (14)	219 ± 20 (9)	222 ± 25 (11)
Distance to nerve ring, % of total oesophagus length	15.2 ± 2.4 (15)	14.7 ± 1.6 (11)	18.9 ± 2.7 (4)
Length of right spicule	101 ± 12 (12)	138 ± 10 (8)	137 ± 15 (11)
Length of left spicule	253 ± 34 (13)	344 ± 30 (9)	354 ± 40 (11)
Length of tail	58 ± 7 (12)	57 ± 7 (12)	73 ± 9 (13)

In caudal region, lateral alae differing in shape and size: left ala larger and more inflated (Fig. 3C). *Area rugosa* present on ventral side of body in posterior part, consisting of rounded bosses arranged in somewhat irregular transverse rows (Fig. 3D). Bosses not reaching cloacal region posteriorly (Fig. 2A). Single cuticular elevated plaque present anterior to cloacal aperture, often slightly shifted to right side. Tail short, conical, with rounded tip, 47–76 [55] long. In most specimens, five pairs of ventro-lateral genital papillae present: four pairs of large and rounded papillae (one pre-cloacal and three post-cloacal) and one pair of smaller sessile papillae located closer to posterior end (Fig. 2D). Papillae on left and right side not always symmetrical in position (Fig. 2E). In some specimens, number of papillae, on at least one side, reduced to four (Fig. 2F and G), or increased to six on one or both sides (Fig. 2H and I) due to presence of additional pre- and/or post-cloacal papillae. Phasmids small, subterminal. Spicules unequal and dissimilar (Fig. 2A). Right spicule short and robust, 72–127 [105] long, slightly curved ventrally, with funnel-shaped proximal end separated from spicule body by apparent constriction (Fig. 2B). Distal end of spicule rounded. Left spicule longer and thinner, 196–314 [287] long, curved ventrally, alate, with rough surface of body and smooth blade; ala almost reaching distal end of spicule (Fig. 2C). Proximal part of spicule not distinctly separated from spicule body.

Females (30 specimens) Table 3. Body 34–68 [52] mm long, 165–313 [185], 311–482 [426] and 306–552 [433] wide at nerve ring, oesophageal-intestinal junction and mid-body length, respectively. Lateral alae beginning at 70–221 [112] from anterior end. Buccal capsule 5–7 [5] long, 4–9 [8] wide. Muscular portion of oesophagus 267–483 [326] long, 31–49 [46], 28–51 [44] and 33–61 [41] wide at anterior, mid-length and posterior level, respectively. Glandular portion of oesophagus 1413–2421 [1817] long, 41–93 [59], 98–205 [115] and 55–164 [75] wide at anterior, mid-length and posterior level, respectively. Total length of oesophagus 1784–2733 [2143], or 3.1–5.7 [4.2] % of body length. Nerve ring situated at 231–373 [261] from anterior end; this distance corresponding to 10.4–20.0 [12.2] % of total oesophagus length.

**Table 3:** Metric characters of females of *Neofoleyellides* spp. from South-African amphibians; measurements are presented as mean ± SD and coefficient of variation (in %) in parentheses.

Character	*N. steyni* n. sp. (N = 30)	*N. martini* n. sp. (N = 30)	*N. boerewors* (after Netherlands et al., 2020)
Body length, mm	53 ± 8 (16)	30 ± 6 (18)	50 ± 16 (32)
Body width at nerve ring	189 ± 34 (18	171 ± 13 (8)	158 ± 24 (15)
Body width at oesophago-intestinal junction	407 ± 42 (10)	372 ± 43 (12)	291 ± 79 (27)
Body width at mid-body	440 ± 63 (14)	409 ± 58 (14)	330 ± 89 (27)
Distance from anterior extremity to anterior end of lateral alae	135 ± 45 (33)	105 ± 28 (27)	not measured
Length of buccal capsule	6 ± 1 (12)	–	4 ± 1 (24)
Width of buccal capsule	7 ± 1 (12)	–	10 ± 2 (22)
Length of muscular portion of oesophagus	375 ± 57 (15)	297 ± 51 (17)	320 ± 61 (19)
Muscular oesophagus width in anterior part	39 ± 5 (13)	35 ± 4 (13)	40 ± 6 (15)
Muscular oesophagus width at mid-length	44 ± 6 (13)	33 ± 4 (11)	34 ± 5 (15)
Muscular oesophagus width in posterior part	43 ± 7 (16)	32 ± 4 (13)	45± (17)
Length of glandular portion of oesophagus	1692 ± 248 (15)	1679 ± 174 (10)	1147 ± 313 (27)
Glandular oesophagus width in anterior part	65 ± 13 (20)	58 ± 11 (18)	74 ± 16 (22)
Glandular oesophagus width at mid-length	143 ± 29 (20)	105 ± 14 (13)	117 ± 30 (25)
Glandular oesophagus width in posterior part	98 ± 26 (27)	74 ± 13 (18)	104 ± 33 (31)
Total length of oesophagus	2067 ± 240 (12)	1976 ± 185 (9)	1467 ± 355 (24)
Total length of oesophagus, % of body length	4.0 ± 1.0 (18)	6.7 ± 1.2 (18)	3.1 ± 0.6 (21)
Distance to nerve ring	288 ± 38 (13)	256 ± 39 (15)	249 ± 56 (23)
Distance to nerve ring, % of total oesophagus length	14.1 ± 2.0 (16)	13.0 ± 1.9 (15)	17.5 ± 4.0 (23)
Distance to vulva	789 ± 145 (18)	1006 ± 185 (18)	1234 ± 266 (22)
Distance to vulva, % of total oesophagus length	38.5 ± 7.6 (20)	50.7 ± 7.2 (14)	83.4 ± 9.9 (12)
Distance to vulva, % of body length	1.5 ± 0.2 (17)	3.4 ± 0.5 (14)	2.9 ± 0.6 (24)
Length of tail	168 ± 39 (23)	105 ± 21 (20)	274 ± 139 (51)

Vulva not salient, transverse, located at level of anterior part of glandular oesophagus (Fig. 1A), at 508–1090 [752] from anterior end of body, this distance corresponding to 1.0–2.1 [1.5] % of body length and 24.3–56.0 [35.1] % of total oesophagus length. Vagina short, globular. *Vagina vera* infundibuliform, lined with thin cuticle. *Vagina uterina* with thick cuticular lining, curved, connected to thick-walled ovejector. Ovejector directed posteriorly, then forming bends and loops along and around oesophagus (Fig. 1A). Uteri and ovaries forming numerous loops and filling whole body of female, not reaching caudal region. Intestine narrow in posterior part. Rectum thin, short, cuticularised. Tail gradually tapering, with rounded tip (Figs. 1I), 101–277 [153] long.

Microfilariae (30 specimens). Sheath often visible around posterior part of microfilaria. Body short, gradually tapering in posterior half (Fig. 1, Fig. 7A) and 70–112 long and 3–7 wide. Anterior end rounded, posterior end pointed. Cephalic hook minute. Crown of about 20 short cuticular ridges present on anterior extremity. Densely arranged chromatin, staining dark purple with Giemsa-stain (Fig. 7A).

Remarks. The new species was assigned to the genus *Neofoleyellides* based on the morphology of apical extremity: oral opening small and surrounded by two lateral parastomal structures, 2 minute pore-like amphids, two small internal lateral papillae and two enlarged external sublateral papillae; in males: presence of caudal alae, wide plaque, and unequal spicules; in females: vulva situated at level of oesophagus; microfilariae with sheath (Netherlands et al., 2020).

*Neofoleyellides steyni* n. sp. can be distinguished from *N. boerewors* by asymmetrical caudal alae in males and the asymmetry in number and position of genital papillae, contrary to narrow and similar alae and four papillae on each side in *N. boerewors*. Two species have different position of the vulva in females: at level of anterior end of the glandular portion of the oesophagus in *N. steyni* n. sp. (24–56% of total oesophagus length) *vs* closer to posterior end of the glandular portion of the oesophagus (68–103% of total oesophagus length) in *N. boerewors*. The shape of the glandular portion of the oesophagus in both sexes is also different in the two species: glandular oesophagus of *N*. *steyni* n. sp. has maximum width at level of the border between the first and second thirds, and narrowing posterior third while glandular oesophagus in *N. boerewors* is evenly widened, with maximum width at posterior end.

*Neofoleyellides steyni* n. sp. clearly differs from *N. martini* n. sp. in morphology of caudal alae in males, position of the vulva and morphology of microfilariae (see below).

**Family Onchocercidae** Leiper, 1911

**Subfamily Waltonellinae** Bain et Prod’Hon, 1974.

**Genus *Neofoleyellides*** Netherlands, Svitin, Smit et Du Preez, 2020.

*Neofoleyellides martini* n. sp.

*Type-host*: *Leptopelis natalensis* (Smith, 1849) (Amphibia: Anura: Arthroleptidae).

*Site of infection*: body cavity, subcutaneous, pericardium.

*Type-locality*: Alfred Park, Pinetown, KwaZulu-Natal Province, South Africa. Coordinates: 29°47′30.4″S 30°52′06.0″E.

Type-specimens: Holotype (NMB P739), allotype (NMB P740) and 20 paratypes (NMB P741), deposited in the Parasitic Worm Collection, National Museum, Charles Street, Bloemfontein, South Africa.

*Infection parameters*: All of eight dissected amphibians were infected with 3–54 (average 15) nematodes.

*Representative DNA sequences*: 18S [MW599275], COI [MW774895].

*ZooBank registration*: To comply with the regulations set out in article 8.5 of the amended 2012 version of the International Code of Zoological Nomenclature (ICZN, 2012), details of the new species have been submitted to ZooBank. The Life Science Identifier (LSID) for *Neofoleyellides martini* n. sp. is urn:lsid:zoobank.org:act:[30C787D3-9B0A-4B47-A32B-1AADE504C855].

*Etymology*: The species is named after Henry Martin (close family friend to author ECN), who provided support and accommodation for the team during several sampling trips to the area and assisted in the initial discovery of this species.

**Fig. 4:**
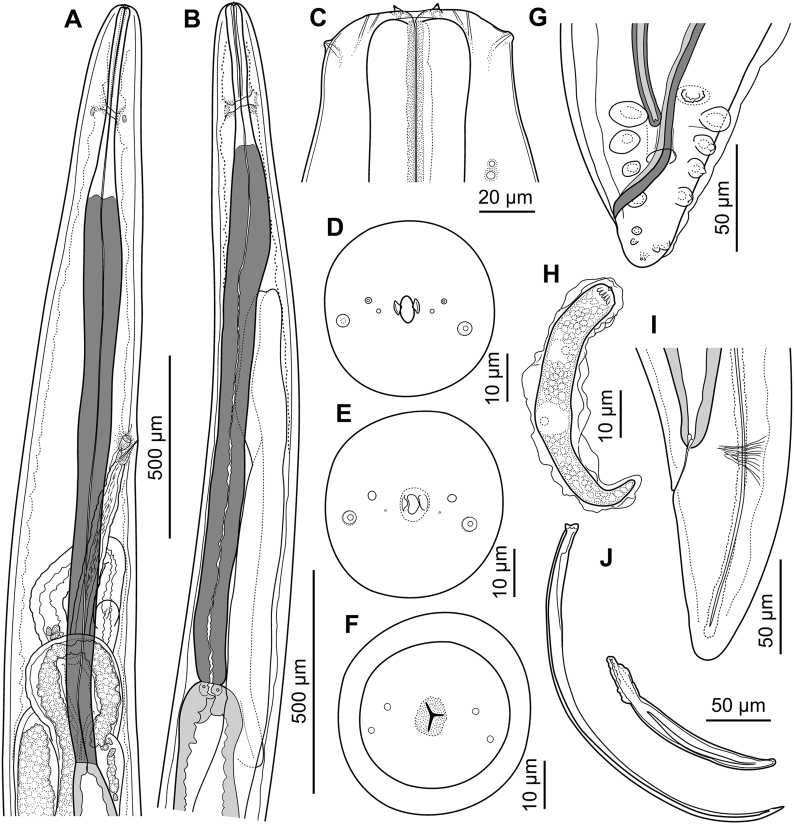
*Neofoleyellides martini* n. sp. from *Leptopelis natalensis* (Smith, 1849), line drawings. A – fragment of body at anterior end, female, lateral view; B – fragment of body at anterior end, male, lateral view; C – anterior extremity, female, lateral view; D–F – anterior extremity, female, apical view, optical sections at different depth of focus; G – posterior end of body, male, ventral view; H – microfilaria; I – posterior end of body, female, lateral view; J – spicules, lateral view.

**Fig. 5:**
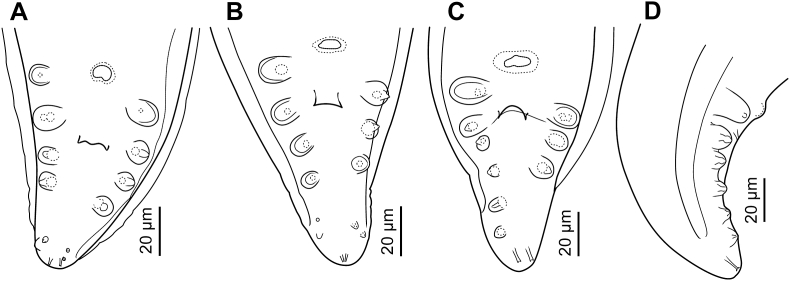
*Neofoleyellides martini* n. sp. from *Leptopelis natalensis* (Smith, 1849), line drawings. A–D – posterior end of body, male, ventral view, variations of the arrangements of caudal papillae.

**Fig. 6:**
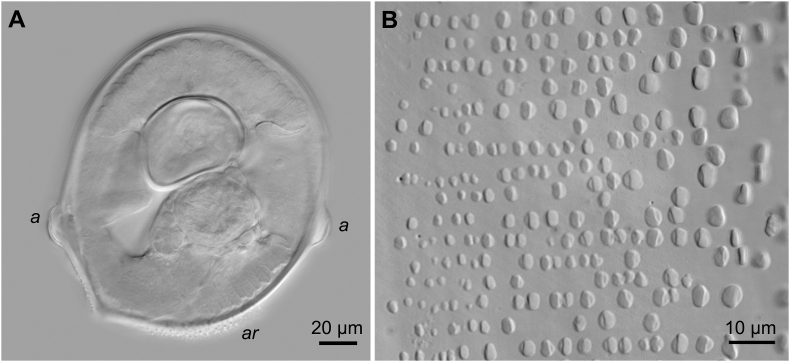
*Neofoleyellides martini* n. sp. from *Leptopelis natalensis* (Smith, 1849), photomicrographs. A – transverse section at posterior end of body, male, a – ala; B – area rugosa.

Description (Fig. 4, Fig. 5, Fig. 6).

General. Body thread-like, tapering on both extremities (Fig. 4A, B, G, I). Females about 2–3 times longer than males. Anterior end rounded. Body cuticle finely transversely striated. Lateral alae present in both sexes, beginning at level of mid-length of muscular oesophagus, almost reaching tail tip posteriorly. Alae narrow, triangular in transverse sections along most of body length, slightly inflated and folded in anterior and posterior regions.

Oral opening oval, elongated dorso-ventrally (Fig. 4D). Two small lateral parastomal structures beside oral opening (Fig. 4D and E). One pair of large cephalic papillae with rounded tips located sub-laterally, slightly shifted to ventral side; one pair of smaller papillae, flattened and less distinct, located laterally closer to oral opening; amphidial openings pore-shaped, distinct, sub-lateral in position, slightly shifted to dorsal side (Fig. 4 C–F). Buccal capsule inconspicuous.

Muscular oesophagus short and narrow, rounded on anterior end, widening posteriorly (Fig. 4A and B). Lumen of muscular oesophagus tri-radiate (Fig. 4F). Border between glandular and muscular oesophagus indistinct. Glandular oesophagus wider and approximately 5–6 times longer than muscular oesophagus, its anterior half gradually widening posteriorly, posterior third narrowing (Fig. 4A and B). Nerve ring at mid-length of muscular oesophagus or slightly posterior to it.

Males (30 specimens) Table 2. Body 14–19 [16] mm long, 113–146 [116], 202–263 [256] and 214–292 [235] wide at nerve ring, oesophageal-intestinal junction and mid-body, respectively. Posterior part of body corkscrew-shaped. Lateral alae beginning at 42–175 [97] from anterior end. Muscular portion of oesophagus 203–308 [225] long, 24–40 [36], 29–51 [35] and 27–41 [29] wide at anterior, mid-length and posterior level, respectively. Glandular portion of oesophagus 972–1607 [1201] long, 42–79 [45], 74–125 [89] and 51–97 [80] wide at anterior, mid-length and posterior level, respectively. Total length of oesophagus 1237–1862 [1426], or 7.5–11.7 [8.7] % of body length. Nerve ring located at 186–258 [191] from anterior end, this distance corresponding to 11.3–18.7 [13.4] % of total oesophagus length. Anterior part of testis narrow, forming loops and bends in region of glandular oesophagus (Fig. 4B). Posterior to oesophago-intestinal junction, testis wide and straight. Ejaculatory duct narrower than testis, with funnel-shaped proximal part.

In caudal area, lateral alae equal, slightly inflated, semi-spherical on transverse section (Fig. 6A). *Area rugosa* present on ventral side of body in posterior part, consisting of rounded bosses arranged in somewhat irregular transverse rows (Fig. 6B). Bosses not reaching cloacal region posteriorly. Single cuticular elevated plaque present anterior to cloacal aperture. Tail short, conical, with rounded tip, 41–76 [55] long. Number and position of genital papillae somewhat variable. In most males (16 out of 30), 12 ventrolateral papillae arranged in two groups: four pairs of large, mammiform papillae in anterior group, of them one pair distinctly precloacal, one pair pre- or ad-cloacal, and two pairs post-cloacal, and two pairs of small papillae in posterior group, closer to posterior extremity (Fig. 4, Fig. 5A). In some specimens (12 out of 30), one papilla in anterior-most pair absent on either side (Fig. 5B). In few specimens, absence of anterior-most papilla accompanied with absence of both posterior papillae on same side, while remaining posterior papillae enlarged and located closer to anterior group (Fig. 5C and D). Phasmids pore-like, subterminal (Figs. 4G, 5A–D). Spicules unequal and dissimilar (Fig. 4J). Right spicule short and robust, 113–159 [140] long, slightly curved ventrally, with funnel-shaped proximal end separated from spicule body by apparent constriction. Distal end of spicule rounded. Left spicule longer and thinner, 275–399 [358] long, curved ventrally, with rough surface of body and smooth blade with sharpened tip. Proximal part of spicule not distinctly separated from spicule body.

Females (30 specimens) Table 3. Body 17–37 [37] mm long, 146–197 [197], 266–468 [468] and 289–551 [447] wide at nerve ring, oesophageal-intestinal junction and mid-body length, respectively. Lateral alae beginning at 65–199 [95] from anterior end. Muscular portion of oesophagus 230–409 [409] long, 24–44 [43], 27–42 [38] and 27–42 [33] wide at anterior, mid-length and posterior level, respectively. Glandular portion of oesophagus 1319–2076 [2076] long, 39–77 [66], 75–142 [110] and 50–113 [82] wide at anterior, mid-length and posterior level, respectively. Total length of oesophagus 1670–2485 [2485], or 3.1–5.7 [4.2] % of body length. Nerve ring situated at 231–373 [261] from anterior end, this distance corresponding to 5.0–10.4 [6.7] % of total oesophagus length.

Vulva not salient, transverse, located at level of oesophagus mid-length (Fig. 4A), at 629–1340 [12,251] from anterior end of body, this distance corresponding to 2.6–4.4 [3.4] % of body length and 36.5–63.5 [50.3] % of total oesophagus length. Vagina short, globular. *Vagina vera* infundibuliform, lined with thin cuticle. *Vagina uterina* with thick cuticular lining, curved, connected to thick-walled ovejector. Ovejector directed posteriorly, then forming bends and loops along and around oesophagus (Fig. 4A). Uteri and ovaries forming numerous loops and filling whole body of female, not reaching caudal region. Intestine narrow in posterior part. Rectum thin, short, cuticularised. Tail gradually tapering, with rounded tip (Figs. 4I), 51–172 [92] long.

**Fig. 7:**
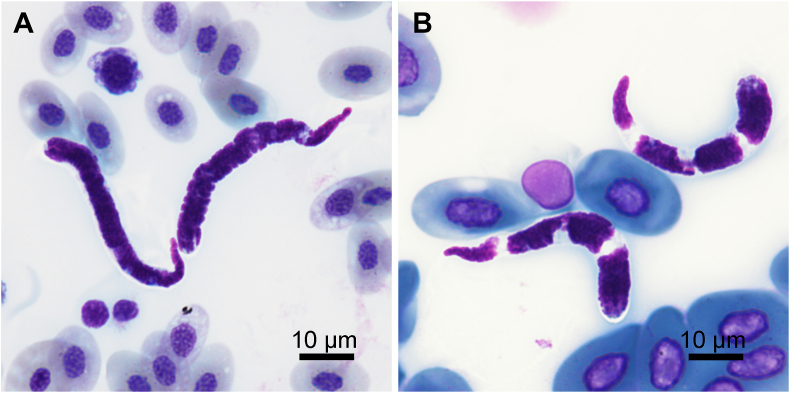
Stained microfilariae from amphibian blood. A – *Neofoleyellides steyni* n. sp. from *Amietia delalandii* (Duméril et Bibron, 1841); B – *Neofoleyellides martini* n. sp. from *Leptopelis natalensis* (Smith, 1849).

Microfilariae (30 specimens). Sheath present as loose envelope around whole body of larva. Body short and stout, rounded on anterior end, tapering in posterior half, 51–79 long and 5–9 wide (Fig. 4, Fig. 7B). Densely arranged chromatin, staining dark purple to pink with Giemsa-stain, slight dilatation apparent just posterior to body mid-region due to presence of large translucent G1 cell (Fig. 4, Fig. 7B). Cephalic hook and crown of 10–15 short cuticular ridges on apical surface.

Remarks. The new species was assigned to the genus *Neofoleyellides* based on morphology of apical extremity: oral opening small and surrounded by two lateral parastomal structures, 2 minute pore-like amphids, two small internal lateral papillae and two enlarged external sublateral papillae; in males: presence of caudal alae, wide plaque, and unequal spicules; in females: vulva situated at level of oesophagus; microfilariae with sheath (Netherlands et al., 2020).

*Neofoleyellides martini* n. sp. can be differentiated from *N. boerewors* by arrangement of caudal papillae in males: often asymmetrical, with first pair pre-cloacal in *N. martini* n. sp. contrary to symmetrical with first pair ad-cloacal in *N. boerewors*. Females of *N. martini* n. sp. have vulva located near oesophagus mid-length (37–64% of oesophagus length) in contrast to females of *N. boerewors* having vulva at level close to oesophagus posterior end (68–103%).

*Neofoleyellides martini* n. sp. differs from *N. steyni* n. sp. in the shape of male caudal alae, relative position of vulva in females and morphology of microfilariae. In males of *N. martini* sp. n., the caudal alae are narrow and symmetrical, while in *N. steyni* n. sp., the left ala is prominently larger than the right one. In females of *N. martini* n. sp., the vulva is usually located more posteriorly (37–64% of total oesophagus length; 3–4% of body length) than in females of *N. steyni* n. sp. (24–56% of total oesophagus length; 1–2% of body length), though this character is variable and the values are overlapping in both species. The microfilariae in *N. martin* n. sp. are shorter (51–79 long), with large G1 cell, while in *N. steyni* n. sp. microfilariae are longer (70–112), with less conspicuous G1 cell.

### 3.2 Phylogenetic analysis

**Fig. 8:**
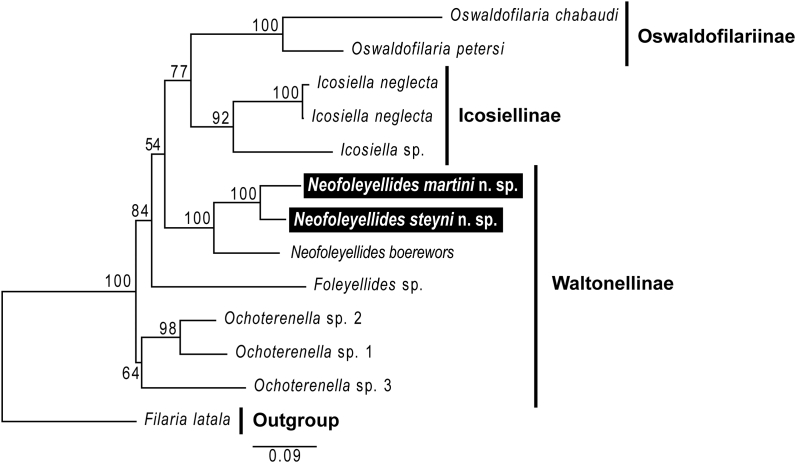
Phylogeny of selected amphibian and reptilian filarial nematodes from the family Onchocercidae. Phylogram based on partitioned and concatenated datasets of 18S rDNA, and COI mtDNA sequences using Maximum Likelihood. *Filaria latala* (GenBank Accession numbers – 18S: KP760135 and COI: KP760186] was chosen as the outgroup. The total length of datasets is 1293 nucleotides, containing 11 taxa. The scale bar represents 0.09 nucleotide substitutions per site.

Sequence amplicons of between 815 and 820 nt of the 18S rRNA gene and 649 and 692 nt of the COI gene were derived from adult stages collected in the body cavity of the hosts *A. delalandii* and *L. natalensis*. In the present study's phylogenetic analysis, species of *Neofoleyellides* are monophyletic and sister to monophyletic group consisting of *Icosiella* spp. and *Oswaldofilaria* spp. (Fig. 8). *Foleyellides* sp. was recovered as a sister taxon to *Neofoleyellides*, however, this is based solely on the COI genetic marker as no 18S rDNA sequences are available for this species. Furthermore, species of *Ochoterenella* are the earliest diverging lineages of the ingroup analysed. The phylogenetic tree topology as well as pairwise analyses based on 18S rRNA and COI alignments (Table 4) showed closer relationships between *N. steyni* n. sp. and *N. martini* n. sp. and more distant relation of both species to *N. boerewors*.

**Table 4:** Pairwise analyses of three species of *Neofoleyellides* based on 18S rDNA (below diagonal) and COI mtDNA (above diagonal) alignments; differences are presented as percentage and number of nucleotides in parentheses.

Species	*N. boerewors*	*N. steyni*	*N. martini*
*N. boerewors*	–	11.7 (52)	13.8 (61)
*N. steyni n. sp.*	2.4 (18)	–	7.7 (34)
*N. martini n. sp.*	2.1 (16)	0.4 (3)	–

## 4 Discussion

The number and position of cephalic structures (two large sublateral external papillae, two small lateral internal papillae, sublateral amphids), the shape of lateral alae (continuous along the body in males and females), and apical morphology of microfilariae (a crown of short radial cuticular ridges) in *N*. *steyni* n. sp. and *N. martini* n. sp. conform morphologically to *N. boerewors*, confirming the diagnosis of the previously monotypic genus *Neofoleyellides*. The most remarkable feature of the genus is the number and arrangement of papillae on anterior extremity. To the best of our knowledge, within the family Onchocercidae only species of *Splendidofilaria* Skrjabin, 1923 share characteristics such as a reduced number of papillae (four instead of eight or more) and bilateral symmetry (Anderson, 2009; Koch and Huizinga, 1971). Members of *Splendidofilaria* are parasitic in birds and belong to a lineage in the Onchocercidae that is distantly related to the subfamily Waltonellinae (Lefoulon et al., 2015; Netherlands et al., 2020). Therefore, we assume that morphological similarities in the apical end between *Neofoleyellides* and *Splendidofilaria* are a result of convergent evolution. Unfortunately, only short fragments of partial 18S rDNA and COI sequences of *Splendidofilaria* sp. are available in GenBank for comparison and could not be included in phylogenetic or pairwise analyses.

At species level, morphological differences between *Neofoleyellides* spp. were found in the morphology of the oesophagus in both sexes of adult nematodes, morphology of caudal alae in males, and morphology of microfilaria. Also, all three species of the genus are significantly different in the relative position of vulva (approximate probabilities <0.0001 in Tukey HSD test between all species).

All three species of *Neofoleyellides* appear to be host-specific and currently known from only one or, in case of *N. boerwors*, two closely related host species. Moreover, at the same localities where species of *Neofoleyellides* were found, about 35 species of amphibians were screened for parasites and no filarial nematodes were recovered (our unpublished data). In two of the most heavily infected common river frogs (34 and 54 nematodes), we found three male specimens of *Neofoleyellides* sp. with narrow caudal alae and evenly widened glandular portion of oesophagus that correspond to the description of *N. boerewors*. Unfortunately, all these specimens were exposed to lactophenol, preventing quality DNA from being extracted and causing molecular sequencing to fail. Moreover, in the same area we have screened 12 guttural toads (*Sclerophys gutturalis*) and four eastern olive toads (*S. garmani*) that are typical hosts of *N. boerewors* and none of them were infected with filarial nematodes (unpublished data). Therefore, we prefer not to assign the atypical male specimens to *N. boerewors* and not report *A. delalandii* as a new host for this species until further studies on host specificity of this group is performed.

The influence of species of *Neofoleyellides* on their hosts' health was not specially studied, although some pathological changes in frogs were observed. One of the tree frogs looked seemed weak and had an unusually grey coloured when collected. During the dissection 12 nematodes were recovered dead in body cavity and subcutaneously, causing inflammation and the spleen to be excessively enlarged (size of the spleen was almost as same as the liver). Another tree frog specimen was found with paralysed hind legs after several days in captivity. During the dissection a mass of nematodes (more than 10 specimens) was recovered from the sacral spine pressuring the hind leg nerves. However, the majority of infected specimens of both frog species did not show any notable changes, even in cases of severe infection (more than 50 nematodes) or the presence of several nematodes found in the pericardium.

Phylogenetic analysis based on concatenated 18S rRNA and COI gene sequences, suggest members of the Waltonellinae are non-monophyletic, with species of *Neofoleyellides* forming a sister group to monophyletic Icosiellinae and Oswaldofilariinae. These findings were similar to Netherlands et al. (2020) based on the same markers. Phylogenetic analysis by Lefoulon et al. (2015) based on seven genetic markers (18S rRNA, 28S rRNA, MyoHC, rbp1, hsp70 and COI, 12S rDNA) found filariae of Oswaldofilariinae, Icosiellinae and Waltonellinae formed a well-supported clade sister to all other taxa studied within the Onchocercidae. Although the present study has added molecular data (18S rRNA and COI) for the two new species of *Neofoleyellides* within Waltonellinae, obtaining data on additional genetic markers for these species may provide better resolution for these early diverging filariae from amphibian hosts.

The common river frog, *A. delalndii* is a widespread species across southern Africa (Du Preez and Carruthers, 2009). The frog is commonly infected with nematodes and seven species were reported in several studies (Baker, 1987; Halajian et al., 2013; Svitin et al., 2017). Despite the parasites of *A. delalandii* were studied from throughout South Africa including Limpopo Province (Halajan et al., 2013), neither adult filarial nematodes nor microfilariae have been previously reported from this host. The locality of *N. steyni* n. sp. reported herein may represent the southern margin of its distribution or might be a result of the vector species distribution range. Although southern Africa has a high diversity of frog species (more than 300 species (Channing and Rodel, 2019)), to date only four amphibian species are confirmed to be infected with species of *Neofoleyellides*. Based on the current study it is clear that there is still much data that needs to be gathered before we come to understanding of the diversity and distribution of filarial nematodes in amphibian from southern Africa.

## Funding

The study was financially supported by the South African National Biodiversity Institute (SANBI) and the 10.13039/501100001321National Research Foundation (10.13039/501100001321NRF), Grant No: 98114; 120782 and 129114. Opinions expressed and conclusion arrived at, are those of the authors and are not necessarily to be attributed to the funding bodies. The financial assistance of the 10.13039/501100001321NRF towards 10.13039/100015589ECN, who is supported by DSI/10.13039/501100001321NRF Innovation Postdoctoral Fellowship (Grant UID: 129,669), is also hereby acknowledged.

## Declaration of competing interest

Authors declare that they have no conflict of interest.
